# Towards developing an operational Indian ocean dipole warning system for Southeast Asia

**DOI:** 10.1038/s41598-025-99261-9

**Published:** 2025-04-27

**Authors:** Shipra Jain, Thea Turkington, Wee Leng Tan, Chen Schwartz, Adam A. Scaife, Theodore G. Shepherd

**Affiliations:** 1 Department of Risk and Disaster Reduction, University College London, London, UK; 2 Centre for Climate Research Singapore, Meteorological Service Singapore, Singapore, Singapore; 3 Met Office Hadley Centre, Exeter, UK; 4 Faculty of Environment, Science and Economy, University of Exeter, Exeter, UK; 5 Department of Meteorology, University of Reading, Reading, UK; 6 Jülich Supercomputing Centre, Forschungszentrum Jülich, Jülich, Germany

**Keywords:** Indian ocean dipole, Climate variability, Climate prediction, Multimodels, Warnings, Atmospheric science, Climate-change impacts, Projection and prediction

## Abstract

Two strong positive Indian Ocean Dipole (IOD) events in 2019 and 2023 led to multiple disasters over Southeast Asia, highlighting the need for warnings of IOD events. This paper presents a stock-take of the current criteria for IOD monitoring and prediction and describes the development of an IOD warning system for Southeast Asia. We examined how subjective choices such as observational datasets, baseline periods, and time averaging affect IOD event identification. Our findings indicate that the choice of sea-surface temperature dataset and time averaging (monthly vs. 3-monthly mean) lead to marked differences in the Dipole Mode Index (DMI), the index used for the monitoring and prediction of IOD events, and hence between various centers on IOD state. The southern Maritime Continent can experience the impact of the IOD on rainfall even when the IOD has not met the current operational criterion, suggesting a need for an impact-based threshold for the IOD. We assess the skill of models in capturing the strength and phase of the IOD and report errors in IOD predictions. While most models are skillful in capturing the active phase of the IOD, many models have an overactive IOD strength. Calibration of DMI-based monitoring products is therefore recommended for the most skilful IOD predictions. Finally, we describe an objective standard operating procedure to assist climate forecasters in issuing timely alerts of IOD events.

**Supplementary Information:**

The online version contains supplementary material available at 10.1038/s41598-025-99261-9.

## Introduction

Southeast Asia suffers from multiple environmental disasters each year. The ASEAN Disaster Information Network (ADINet) reported over 6000 disasters in Southeast Asia from 2012 to date, with ~ 93% of total disasters consisting of hydro-meteorological hazards, including floods, droughts, and storms (http://www.adinet.ahacentre.org). The rainfall-related hazards, particularly flood and drought, over this region are closely connected to the state of the world’s ocean, especially the El Niño-Southern Oscillation in the tropical Pacific and the Indian Ocean Dipole (IOD) in the tropical Indian Ocean basin^1^. These oscillations are also the key drivers of climate variability from monthly to interannual timescales for many tropical regions, including Africa, Australia, South Asia, and Southeast Asia^2^.

In 2019, a strong positive IOD event occurred, and it was one of the most extreme IODs in the historical observational record. This event led to multiple cascading hazards in the region. For instance, Indonesia experienced a severe and widespread drought, with many locations reporting a dry spell lasting several weeks^3,4^. The Mekong River, which is one of the longest rivers in East and Southeast Asia, was at its historic low in the last 100 years or so, affecting fisheries, agriculture, freshwater availability, tourism, biodiversity, employment, and the economy of multiple countries in this region^5^. Singapore recorded an unusual dry spells in July-August, with no rain for 16 consecutive days^6^. There were widespread forest fires in parts of Indonesia, including Sumatra and Borneo Island. The smoke from these fires not only affected the region close to the fire hotspots but was also transported across the hemisphere by large-scale circulation, leading to hazy conditions in areas much farther away from the fires^7^. It resulted in severe air quality and health hazards in some of the most populated cities in Southeast Asia, such as Kuala Lumpur, leading to significant disruptions in daily life, e.g., closure of schools and offices and cancellation of flights.

The record-breaking 2019 IOD event affected not only Southeast Asia but also many neighboring continents. Severe drought and wildfires were experienced over parts of Australia, and heavy rainfall and flooding in East Africa. India reported one of the latest withdrawals of the Indian summer monsoon. This event impacted extreme events as far away as the Atlantic^8,9^. In addition to its widespread atmospheric impacts, this event also impacted biological processes in the Indian Ocean basin, leading to anomalous chlorophyll concentrations^10,11^. After the 2019 event, another extreme positive IOD occurred in 2023 and had far-reaching impacts. As a result, there is now significant interest from the users of climate information in real-time IOD monitoring and outlooks, which would allow them to take proactive steps to manage the impacts, especially in the Southeast Asian countries, which lie near the Indian Ocean basin.

Currently, the regional-level forecasts for Southeast Asia are issued through the ASEAN Climate Outlook Forums (COFs) and the Southeast Asia Regional Climate Centre (SEA-RCC) Network. The national-level forecasts are delivered by national meteorological agencies, specifically tailored to individual countries and sectors. The SEA-RCC Network and the individual national meteorological agencies primarily rely on the products available through the Bureau of Meteorology (Australia) and the Copernicus Climate Data Store (Europe) for IOD monitoring and outlook. While it is indeed convenient to simply use the products currently available through these centers, there are specific challenges in employing these products to address the operational needs of this region.

One of the key challenges in using the products of other operational centers is the difference in the dataset and criterion used for IOD monitoring. The centers also have their preferred, and sometimes in-house, sea-surface temperature (SST) datasets for monitoring IOD, which may or may not be publicly available. The centers also use different baseline climatologies to calculate the Dipole Mode Index (DMI) - the index used to monitor and predict IOD events. Verdon-Kidd (2018)^12^ has previously examined the impact of using different SST observations and baseline climatology on IOD classification. They found that the IOD classification is sensitive to the choice of SST data, which in turn can lead to differences across the centers on the current state or timing of alerts of IOD events. A recent example of such a situation is the 2018 positive IOD event when the Japan Meteorological Agency (JMA) declared a positive IOD event, whereas the other operational centers, including the Bureau of Meteorology (Australia) system, stayed in IOD-neutral conditions. Under such circumstances, it is difficult to determine which center to follow, highlighting the need for an independent product for Southeast Asia. It is also worth noting here that similar factors also initiated the development of the El Niño Watch System for Southeast Asia, which is now operationally used by the SEA-RCC Network to issue regional-level ENSO alerts for Southeast Asia^13^.

Each operational center providing IOD products follows its own schedule for reviewing present conditions, declaring IOD events, and issuing alerts/warnings and outlooks, resulting in varying release times throughout the month. Consequently, using their products implies waiting for their updates to issue an alert for Southeast Asia. Additionally, the presentation of these products (e.g. color scheme or scales) cannot be modified, further limiting the uptake of these products for tailored regional or national needs.

Developing a climate service often requires decision-making on the part of the service producer. For climate drivers, such as the IOD, this includes choosing an appropriate observational or model dataset for the product, a diagnostic that is a good representative of the phenomenon, a baseline climatological period, and criteria that need to be met to identify an IOD event. These choices are inherently subjective and the scientific rationale behind them is often not properly documented. Through this work, we aim to establish good practices for developing operational products and services, as strongly recommended by the World Meteorological Organization^14^. This would enhance the reproducibility and traceability and ensure that future upgrades to the products or the development of similar new products can build on this product, and duplication of effort can be avoided. As the current monthly and seasonal outlooks follow a consensus-based approach through ASEANCOFs, we also propose establishing an objective Standard Operating Procedure (SOP) for issuing regional or national-level IOD alerts. Implementing an objective SOP will help minimize the influence of personal judgments of climate forecasters in issuing alerts, which is also in line with the WMO recommendations^14^.

This paper is organized as follows: We first perform a stock-take of the existing operational IOD monitoring and outlook products. We then assess the impacts of the differences in the operational criteria across the centers on the identification of the IOD events (“Stock-take of operational IOD products”). We then examine the relevance of the DMI threshold that is currently used operationally to identify the IOD events (“Need for impact-based forecasts of the IOD”). For any climate outlook system to be more useful than climatology, the models must have predictive skill for the driver, and it is important for models to correctly capture the amplitude of fluctuations. Therefore, we assess the performance of the latest prediction systems in capturing the phase and strength of the IOD (“Forecasting capability of the current IOD warning system”). Finally, we summarize the details of the IOD Warning System developed for Southeast Asia and outline the SOP for issuing IOD warnings in “Standard operating procedure (SOP) for the IOD warning system”. The key conclusions and future outlook from this study are provided in “Conclusions and future outlook”.

## Data and methodology

The average SST anomalies over the western pole of the Indian Ocean (− 10 to 10 °N, 50 to 70 °E) are referred to as the Western Tropical Indian Ocean (WTIO) index, and over the southeastern pole (− 10 to 0 °N, 90 to 110 °E) are referred to as the South Eastern Tropical Indian Ocean (SETIO) index. The difference in SST anomalies over the WTIO and SETIO is referred to as the Dipole Mode Index (DMI), which is used for the representation of the IOD^1^ in this paper.

The SST analysis in this work is restricted to 1960 onwards, when there is more consistency between the SST datasets^15^, and from 1979 onwards for rainfall, based on the availability of satellite-based rainfall datasets (Table 1). The model hindcasts analyzed in this paper are available only for 1993–2016, and therefore, all analysis using models is restricted to this period. Note that for the UKMO model, January 1993 data is not available, and therefore, all analysis excludes this month. Also, for NCEP, the number of ensemble members varies for each month, i.e., 28 for January and April, 20 for February, and 24 members for the remaining months. The term multi-observational mean refers to the mean of multiple observational datasets.

**Table 1:** The observational and model data used for the analysis.

Variable	Dataset/source	Data type	Period	Resolution	Ensemble members
Sea surface temperature (SST)	COBE HadISST ERSSTv5	Gridded observations	1960–2021	1° × 1° 1° × 1° 2° × 2°	1
Rainfall	CMAP and GPCP-SG_L3_v2.3	Gridded observations	1979–2021	2.5° × 2.5°	1
SST	NCEP CFSv2 ECMWF S6 JMA CPS3 MeteoFrance UK GloSea6	Model hindcasts	1993–2016	1° × 1°	NCEP: 24 UKMO: 28 JMA: 10 ECMWF: 25 MeteoFrance: 25
Wind	ERA5	Reanalysis	1979–2021	0.25° × 0.25°	1
Outgoing longwave radiation (OLR)	NOAA interpolated OLR	Gridded observations	1979–2021	2.5° × 2.5°	1

## Stock-take of operational IOD products

We first collate the details of the operational products and criteria used by various meteorological centers to identify an IOD event (Table 2). In summary, all centers examined here identify IOD years as the absolute value of DMI exceeding the threshold value of 0.4 for a specific time period, however, they use different SST datasets, baseline climatology, and time-averaging (weekly to 3-monthly mean DMI). We also highlight that most centers issue real-time IOD warnings as part of their monthly or seasonal outlook report, alongside the outlooks for other climate drivers and variables. The detailed criterion used for the start and end time of an IOD event and the issue of real-time warning is not always clear and is likely based on the expert judgment of climate forecasters. There are also differences in the criterion used for real-time monitoring of the IOD and identification of IOD events in the historical record, and some centers provide more than one real-time IOD monitoring product using a different baseline climatology.

We examine the sensitivity of the DMI, and hence IOD years, to the key differences noted above, i.e., the choice of SST datasets, baseline climatology, and time-averaging, using a suite of observational SST datasets. We consider 12 different definitions of an IOD event using a combination of three different publicly available SST datasets (COBE, ERSST, HadISST), two different baseline climatological periods (1981–2010 and 1991–2020), and two different choices of time-averaging (monthly and 3-month running mean) for the DMI (Supplementary Table S1). Note that the baseline climatological periods chosen here are in line with World Meteorological Organization (WMO) Climatological Standard Normals (https://community.wmo.int/en/wmo-climatological-normals). The positive IOD (pIOD) years are then defined as those for which monthly mean DMI > 0.4, and negative IOD (nIOD) as those for which monthly mean DMI < − 0.4, for at least three consecutive months (see DMI definition in methods). In addition to the three individual SST datasets, we also examine the pIOD and nIOD years for the average of these datasets (hereafter referred to as multi-observational mean DMI).

**Fig. 1:**
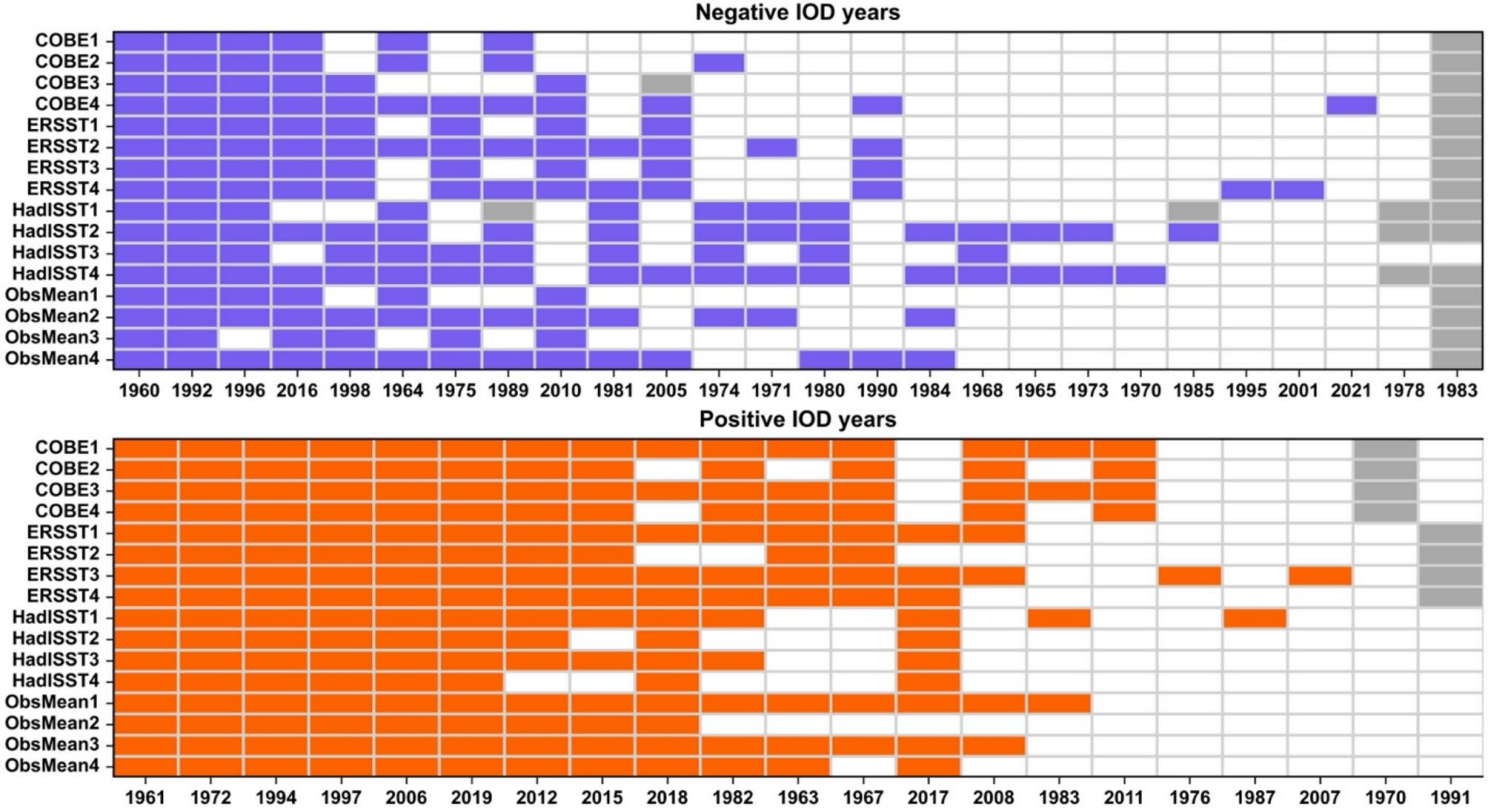
IOD years in observations. List of nIOD (blue) and pIOD (orange) years between June and December identified using 16 different definitions in Table 3. Grey color shows events starting and ending between January and June. On the y-axis, suffix 1 is for 1981–2010 baseline climatology and monthly mean; 2 for 1991–2020 baseline climatology and monthly mean; 3 for 1981–2010 baseline climatology and 3-month running mean; and 4 for 1991–2020 baseline climatology and 3-month running mean. More details are provided in Table S2.

We find that the identification of an IOD event is most sensitive to the SST dataset and time-averaging but relatively less sensitive to the baseline period (Fig. 1). This is consistent with Verdon-Kidd (2018), who also found a significant impact of the SST dataset on IOD classification. We provide a range by which the DMI can differ due to each of these three factors (Fig. S1, Supplementary Material). As a rough estimate, the monthly mean DMI in the observational mean can differ from the individual SST datasets by up to ± 0.6 (Fig. S1). The impact of time-averaging is of similar magnitude, whereas the impact of the baseline climatology is smaller, up to ± 0.3. Some of the differences in DMI across SST datasets can be attributed to the differences in the variability of each dataset. The HadISST dataset shows relatively lower variability compared to COBE and ERSST over almost the whole Indian Ocean basin (see Figs. 4a, S2). However, the largest differences occur over the eastern pole of IOD and, therefore, can potentially contribute more to the differences in the DMI values between the HadISST and the other two datasets. There is also a significant monotonically increasing trend (using the Mann-Kendall test) in the HadISST monthly DMI time series between June and December (trend calculated for each month separately for 1960–2021), whereas no significant trend is found in COBE or ERSST, which may further explain some differences in DMI values. Unlike the variability, no organized differences are noted in the SST trends between the eastern and western poles of the IOD (Fig. S3).

Out of the 21 years identified with a pIOD, only around one-third of the years are identified as pIOD years in all different definitions. For the nIOD, the agreement is even less with only three years out of 26 years meeting the criterion of an nIOD event in all definitions used. The strongest IOD events on record are identified in most datasets, with most disagreements occurring for weak to moderate IOD events. Out of the three SST datasets considered here, there is more agreement between COBE and ERSST, and relatively less agreement between these two datasets and HadISST. Finally, several events appearing as IOD in single SST datasets are filtered out by the multi-observational mean, which thereby provides a more robust estimate of the IOD events (see ObsMean1 to ObsMean4 in Fig. 1).

As mentioned above, there is a significant monotonically increasing trend in the HadISST monthly DMI time series between June and December (trend calculated for each month separately for 1960–2021), whereas no significant trend is found in the multi-observational mean DMI. This highlights another advantage of using a multi-observational mean – it cancels out sporadic multidecadal trends in the DMI time series. We also show in Sect. 5 that seasonal forecasts verify better against multi-observational means than against individual observational datasets.

Despite the differences mentioned above, there are some similarities between all datasets. All datasets show that typically most IOD events occur between June to December, however, there are a few years when the IOD develops before June (e.g. the 1992 nIOD event which started to develop in February-March 1992) or persists beyond December (e.g. the 1997 pIOD event which persisted until January 1998, see Fig. S4 of the Supplementary Material). In addition, there are also some years where the development and decay of an IOD event occurred between January-June (shown by the grey color in Fig. 1). Notably, the nIOD event in 1983, which appeared in Feb–April, is identified in almost all definitions. This year was not preceded or succeeded by an IOD event of the same phase, however, it was preceded by an IOD event of the opposite phase, i.e. pIOD in 1982. The IOD events in the historical record are shown for the multi-observational mean (OBSMEAN2 and OBSMEAN4) in Fig. S4.

**Table 2:** Summary of IOD-related products provided by multiple operational centers. The criteria used to identify an IOD event in real-time (i.e. Before and after 6 months) and in the historical observational record (older than 1 year from the present time) is also provided, wherever available. Data refers to the observational dataset. Note that for this table we only refer to the products that are publicly available through the centers’ website. See Table S1 in supplementary material for the weblink to the products. DMI refers to the dipole mode index, South Eastern Tropical Indian Ocean index (SETIO) refers to the Eastern pole of DMI and Western Tropical Indian Ocean (WTIO) refers to the Western pole of DMI (see methods for definition).

Centre	Data, index and climatology	Details and criterion for real-time event and outlook	Criterion for IOD event in historical record
APEC Climate Centre (APCC)	Index: Monthly mean DMI Data: ERSSTv5 (historical) and OISST (real-time) Climatology: 1991–2020	Provides IOD index time series for the last 6 months and outlook for the next 6 months, does not comment on start or end of the IOD event, or value of the IOD index threshold	Provides IOD index time series from 1948 onwards, does not comment on IOD year
Bureau of Meteorology (BoM)	Index: Weekly DMI Data: Bureau SST dataset and ERSSTv5 (historical) and Bureau SST dataset (real time) Climatology: 1961–1990	Event declaration: Weekly IOD index value exceeding the threshold of ± 0.4 °C for 8 consecutive weeks* (either values have already exceeded the threshold for 8 consecutive weeks, or a current run is strongly anticipated to last at least that long based on current observations and model outlooks) Event start date: first week of the run exceeding the threshold of ± 0.4 °C (i.e. the run of weeks that eventually led to the declaration) Event end declaration: Weekly IOD index value returning to within neutral bounds for 1–2 consecutive weeks, with the end date for an event taken as the last week exceeding the threshold, before the return to neutral* *Leeway employed based on expert judgement	Before 2001, monthly index exceeding the threshold for at least 2 consecutive months between June–December* After 2001, weekly IOD index exceeding the threshold for at least 8 consecutive weeks between June–December* *Leeway employed based on expert judgment
Copernicus Climate Change Service (C3S)	Index: Monthly mean DMI, SETIO, WTIO Data: ERA-5 Climatology:1993–2016	Provides IOD index time series for the last 5 months and outlook for the next 6 months, does not comment on start or end of the IOD event or value of the IOD index threshold	Does not provide information on historical events
Indian National Centre for Ocean Information Services (INCOIS)	Index: Monthly Mean DMI, SETIO, WTIO Data: OISST and INCOIS-GODAS Baseline Period: 1981–2010	Provides IOD index time series for the last 12 months, no information on the outlooks, does not comment on the start or end of the event or value of the IOD index threshold	Does not provide information on historical events
Japan Meteorological Administration (JMA)	Index: Monthly mean and 3-month running mean DMI, SETIO and WTIO Data: COBE-SST2 and MGDSST Baseline Period: 1991–2020 and sliding latest 30-year base period	Provides IOD index time series for multiple time-mean and climatologies, does not comment on the start or end of the IOD event, does not provide outlook, uses a DMI threshold of ± 0.4	Provides IOD index time series for multiple time-mean and climatology from 1950 onwards Provides a list of IOD years/events from 1949 onwards, events/years identified as 3-month running mean DMI exceeding the threshold for at least 3 consecutive months between June–December using 30-year sliding climatology
National Oceanic and Aeronautics Administration (NOAA)	Index: Monthly mean DMI, SETIO and WTIO Data: HadISST1.1 Baseline Period: 1981–2010	Provides IOD index time series, does not provide outlook, does not comment on the start or end of the IOD event or value of the IOD index threshold	Provides IOD index time series from 1870 onwards, does not comment on the IOD year
UKMO	Index: Monthly mean DMI, SETIO and WTIO Data: OSTIA Baseline Period: 1993–2016	Provides IOD index time series for the last 5 months and outlook for the next 6 months, does not comment on start or end of the event or value of the IOD index threshold	Does not provide information on historical events
World Meteorological Organization Lead Centre for Long-Range Forecast Multi-Model Ensembles	Index: Monthly mean DMI Data: NOAA OISST Baseline Period: 1982–2010	Provides IOD index time series for the last 5 months and outlook for the next 6 months, does not comment on start or end of the event or value of the IOD index threshold.	Does not provide information on historical events
JAMSTEC	Index: DMI, Indian Ocean Subtropical Dipole Index (SIOD), and Indian Ocean Basin Mode Index Data: Information not available on website Baseline Period: 1991–2020	Provides IOD index time series for the last 12 months and next 12 months, does not comment on criterion for start or end of the event or value of IOD index threshold	Does not provide information on historical events

## Need for impact-based forecasts of the IOD

Table 2 demonstrates that some operational centers identify an IOD event when the DMI exceeds a threshold of 0.4 for a specific period, typically 3 months. For real-time monitoring, the IOD alert is also generally issued when the DMI exceeds this threshold of 0.4 and is expected to remain above this threshold for a specified period. This threshold is mainly chosen for its statistical relevance—a DMI of 0.4 is approximately equal to one standard deviation in the monthly DMI time series (Fig. 4)—rather than the impact on regional climate. Therefore, here we assess what the threshold of 0.4 implies for rainfall in Southeast Asia.

**Fig. 2:**
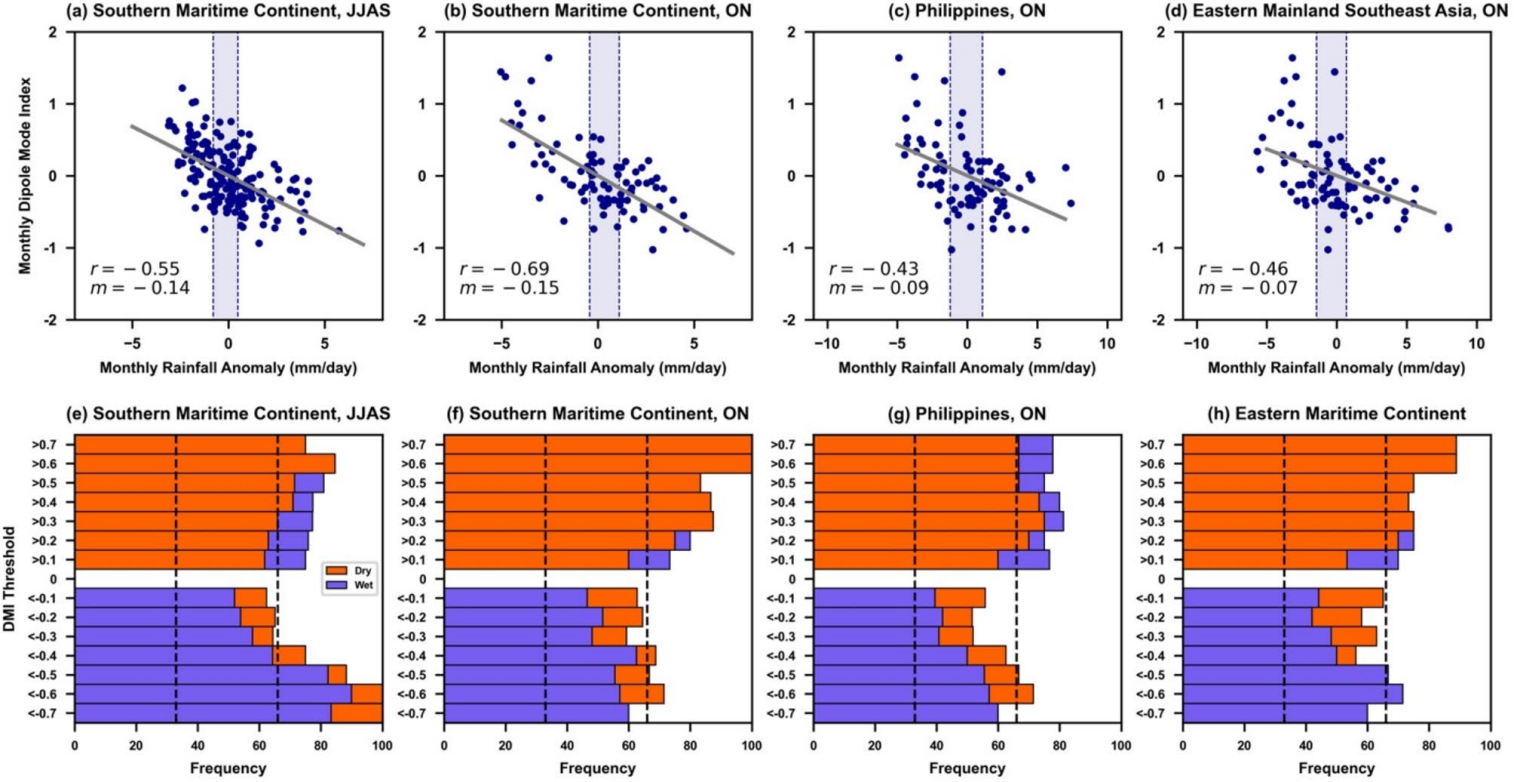
Observed relationship between regional rainfall and IOD phases Spatially-averaged monthly rainfall anomalies and monthly mean DMI for (**a**) Southern Maritime Continent in JJAS, (**b**) Southern Maritime Continent in ON, (**c**) Philippines in ON, and (**d**) Eastern Mainland Southeast Asia in ON. Only the combinations of those regions and seasons are shown which have a significant correlation; the remaining combinations are shown in Fig. S7 (Supplementary Material). The monthly mean DMI used here is the multi-observational mean DMI from COBE, HadISST, and ERSST and monthly rainfall anomalies are the multi-observational rainfall anomalies from the GPCP and CMAP for 1979–2021 (Table 2). Dashed lines in panels (**a**–**d**) show the 33rd and 66th percentile values of the rainfall anomaly distribution, the grey line shows the line of best fit, m is the slope of the line of best fit and r is the Pearson correlation coefficient. (**e**–**h**) percentage of wet months (blue, rainfall anomalies > 66th percentile) and dry months (orange, rainfall anomalies < 33rd percentile) out of total wet, dry, and normal months for each given DMI threshold (see text for details). Black dashed lines show the 33rd and 66th percentiles.

We first identify regions that show spatially coherent rainfall responses to different IOD phases (Figs. S5 and S6 of Supplementary Material). We then plot the scatter diagram for the amplitude of observed DMI and observed rainfall anomalies for the spatially aggregated regions (Fig. 2). During June–July–August–September (JJAS), northern Southeast Asia is wetter than the southern part, owing to the monsoonal circulation and the location of the tropical convergence zone (Figs. S5 and S6). During October–November (ON), the rain band associated with the tropical convergence zone shifts to the equatorial region (− 5 to 5 °N), leading to more rainfall over this region, whereas the regions north and south of the equator are relatively dry. Therefore, here we collate June–September and October–November results separately. The strongest impact of IOD on rainfall is observed over the eastern Indian Ocean and Southern Maritime Continent. This is expected as the Southern Maritime Continent lies at the center of the eastern pole of the IOD. The impact of IOD on this region is persistent and covers both monsoon (JJAS) and inter-monsoon (ON) seasons (Figs. 2a,b, S5 and S6).

The correlation coefficient between the DMI and rainfall anomaly is reasonably high for the Southern Maritime Continent (− 0.55 for JJAS and − 0.69 for ON), suggesting that 30–50% of the variance in rainfall anomalies can be explained by the DMI over this region (Fig. 2a,b). There is also a substantial impact of the IOD on the Philippines (Fig. 2c) and Eastern Mainland Southeast Asia (Fig. 2d) during ON. The DMI and rainfall anomaly correlation for these two regions is also significant (− 0.43 for the Philippines and − 0.46 for Eastern Mainland Southeast Asia). For the remaining regions and seasons, no systematic impact of DMI on rainfall is observed as the correlations are too small (see Fig. S7).

In the observational record, over the Southern Maritime Continent, there were a total of 31 months with DMI > 0.4, out of which 22 months were dry (~ 71%), 7 were normal (~ 23%), and only 2 months were wet (~ 6%) for JJAS (Fig. 2e). This gives the observed frequency (or climatological probability) of a dry, normal and wet month for the historical record given a threshold of DMI > 0.4, compared with a marginal probability of 33%. Thus, there is a higher chance of dry months during pIOD with magnitude > 0.4 and this chance systematically increases with increasing strength of the DMI. For example, given a DMI of 0.6 or above, no wet months were observed in the historical record for this region during JJAS.

The impact of IOD is even stronger during the inter-monsoon season (ON) for this region. For a DMI of strength > 0.3, more than ~ 75% of months are dry whereas the remaining are neutral with apparently no wet month on record (Fig. 2f). This implies that this region can be affected by the IOD even when the DMI has not met the operational criterion (a threshold of DMI > 0.4 is currently used by most operational centers). While the influence of other climate drivers is possible, such as ENSO or MJO, the analysis period contains several months of dry anomalies during pIOD (values between 0 and 0.4) with no ENSO, and the drier conditions are also noted for seasonal means, which negates the impact of MJO. For example, the 2019 positive IOD event did not coincide with an El Niño^8^.

Similar results can be seen for nIOD, though the response of rainfall appears to be relatively weaker for nIOD as compared to pIOD. For example, the observed chance of a dry month during a pIOD > 0.4 is larger than the chance of a wet month during a nIOD <-0.4. Also, the DMI distribution is positively skewed with pIOD events occurring more frequently than nIOD (also see paper^16^, which could also lead to a weaker response of rainfall for nIOD events as compared to pIOD. Note that the sample size for DMI exceeding the threshold of 0.4 is limited due to the limited number of IOD events in the observational record. However, despite the limited sample size, the rainfall shows a response to the strength of the DMI. Similar results are also noted for the northern Philippines and Eastern Mainland Southeast Asia (Fig. 2g,h).

From a climate service perspective, providing a chance of exceeding different DMI thresholds (e.g., chance of DMI exceeding a threshold of 0.3, 0.4, and so on) could, therefore, be useful to take into account the diversity in the impact of IOD on different regions, particularly for the Southern Maritime Continent.

## Forecasting capability of the current IOD warning system

It is important to be aware of the limitations of IOD predictions. Here, we assess five open-access seasonal prediction systems for their ability to capture the phase and strength of IOD events.

**Fig. 3:**
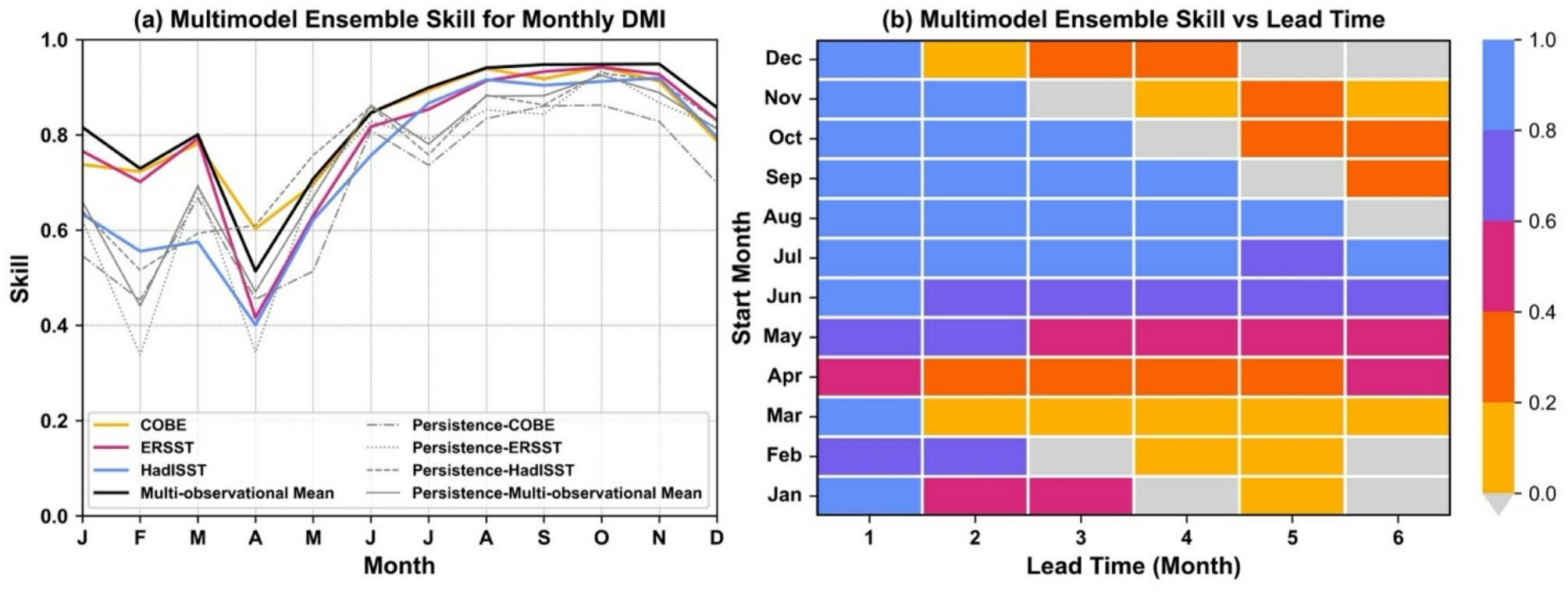
Model skill in predicting the phase of the IOD. (**a**) Multimodel ensemble skill for a lead time of 1 month for the common hindcast period, 1993–2016, using three different observational datasets (colored lines) and multi-observational mean (black line). Multimodel ensemble skill is calculated by averaging the ensemble mean DMI time series of all models, and then correlating it with the observational time series. The lead time of 1 month implies the forecast initialized at the beginning of the month or during the previous month. Grey lines show the persistence for each month for all three observational datasets as well as the multi-observational mean. Persistence here is defined as the anomaly correlation coefficient between the actual DMI anomaly observed during a month with the observed anomalies of the previous month. For example, the December actual DMI anomalies are predicted using the persisted anomaly from November of the same year. The ACC is calculated for the period 1991–2020, the same as the baseline period for which the DMI is calculated, to ensure that the climatological anomalies are close to zero. (**b**) Model skill scores for common hindcast period, 1993–2016, and different lead times. The skill scores are calculated against the multi-observational mean DMI calculated using the 1991–2020 climatological period.

We find that the individual models are very skillful in predicting the phase of the IOD, with models showing skill scores (correlation coefficient) as high as 0.9 for 1-month lead time (perfect skill is 1) (Fig. 3a, Fig. S8), consistent with previous studies^17^. We have also compared the persistence forecast of the DMI with model skill and found that model skill is generally higher than persistence values, particularly during NH autumn and winter, when the IOD is typically present (Fig. 3a). We examined the impact of observational errors on skill scores. The skill scores can vary substantially from January to May when IOD events are typically not present and random noise in observations can lead to differences in the skill scores (Fig. 3a). Models initialized between January and May show relatively lower skill for IOD for all lead times (Fig. 3b). This is due to the low amplitude of DMI for shorter lead times (< 3 months) and a typical reduction in skill for longer lead times (> 3 months). However, note that some studies have demonstrated useful skill, out to 2 years, for the IOD using decadal prediction systems^18^.

Overall, verifying against the mean of different observational datasets (black line) gives better skill scores than using individual observational datasets. However, in contrast, skill using a multimodel ensemble (MME) does not exceed the most skillful single model (Fig. S9). This has also been noted for other tropical regions where using MME or increasing ensemble size usually leads to only a limited improvement in skill^19,20^, and contrasts with extra-tropical prediction where a much larger ensemble and multimodel mean is sometimes needed to gain high skill^21,22^.

Note that the choice of climatological baseline period does not affect the skill scores presented here. The model skill is also lower for the 3-month running mean DMI (i.e., for the next 3 months from the model start time) as compared to the monthly mean DMI, particularly for January to May, as the skill drops with lead time (not shown). There are also some potential limitations of predictability analysis. One of the biggest challenges is the small number of IOD events in the historical record; our results on prediction skill are conditional on the realistic representation of the IOD in observations. The second limitation is that a lot of IOD events coincide with ENSO events, and therefore some of the results that we show (e.g., impact of IOD on rainfall, prediction skill scores) could reflect the impact of another large-scale climate phenomenon^23,24^.

**Fig. 4:**
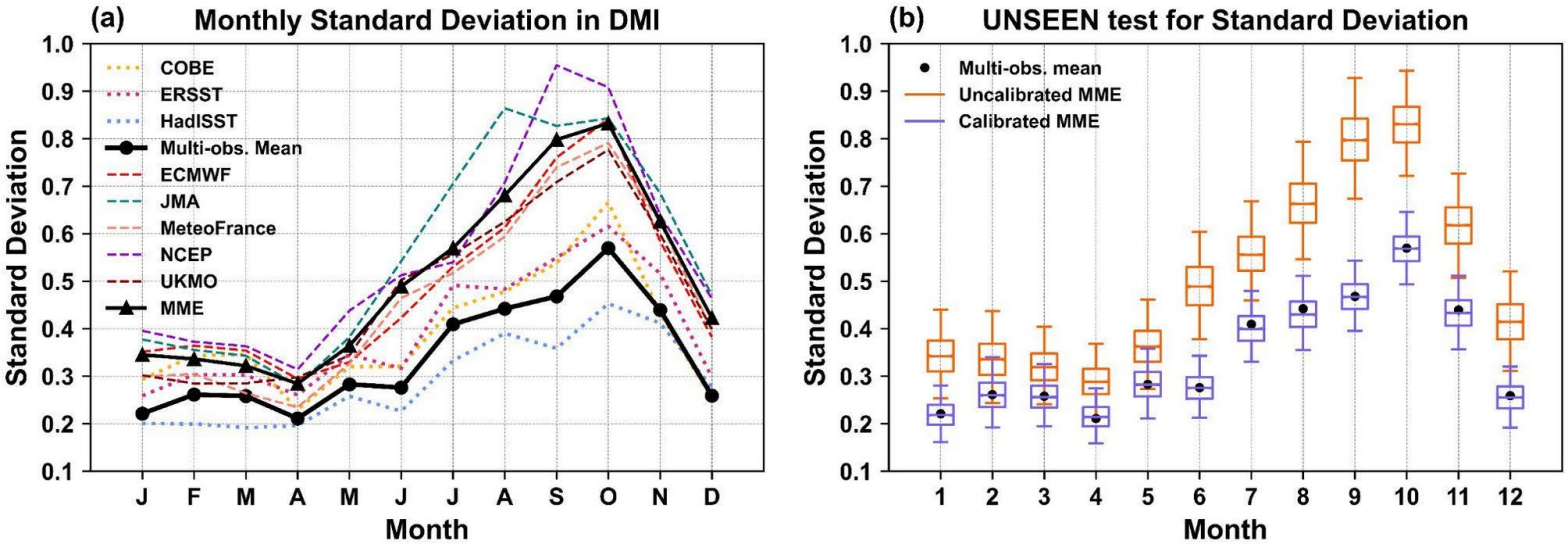
Fidelity of models for IOD strength. (**a**) Standard Deviation (SD) in observed and model DMI time series for the hindcast period (1993–2016). The model DMI is calculated with respect to the hindcast climatology (1993–2016) and observed DMI with respect to the 1991–2020 climatology. For models, the SD of each ensemble member is calculated first, which is subsequently averaged for each model to obtain individual model SD (colored dashed lines) and averaged over all models to obtain MME SD (black line with triangles). Only lead time 1 hindcasts are used for the models. (**b**) SD in multi-observational mean DMI (black circles) and uncalibrated (orange box and whiskers) and calibrated (blue box and whiskers) ‘resampled’ DMI time series from the MME. The distribution is created using 10,000 ‘resampled’ DMI time series, which were obtained by randomly resampling one ensemble member DMI for each calendar year for the model hindcast period (1993–2016). The calibration of DMI time series was done for each month separately by using a multiplicative correction factor, which is defined as the SD in multi-observational mean (circles in Fig. a) divided by the mean of ensemble member SD by pooling all models (triangles in Fig. b). The whiskers denote 2.5 and 97.5 percentiles, and the horizontal line denotes the median SD. Results for individual models are shown in Fig. S7.

We further examine the models’ performance in capturing the strength of the IOD events using the standard deviation (SD) of the DMI as a measure of the IOD strength. Results show that all models have larger SDs than observations for all months of the year (Fig. S10, also see Lu et al. 2018), though there are differences between the observations as well. The SD in COBE and ERSST is larger than in HadISST and therefore closer, though still less than the models’ SD from June to December (Fig. 4a).

Larger SD in models could be due to a systematic error in SSTs in models, which induces a positive IOD-like pattern in the SSTs and precipitation. In some models, these errors are noted to develop shortly after initialization and grow over the forecast period^25^. Past studies have suggested that these errors in SSTs appear to originate due to the excessively strong low-level easterlies over the equatorial Indian Ocean, leading to a shallow thermocline over the eastern Indian Ocean through the Bjerknes feedback and stronger SST gradient between western and eastern equatorial Indian Ocean^17^. This SST error would also lead to a larger SD in the DMI leading to an overactive IOD.

To examine whether the over-active IOD strength in the models is acceptable or if the IOD forecasts need calibration, we performed fidelity tests for the SD using ‘resampled’ time series from models (Fig. 4b, see captions for methods, also see papers^26,27^. We find that all models had an unrealistically over-active IOD during June-December, as the observed SD (black circles in Fig. 4b) lies outside the SD distribution (orange box and whiskers in Fig. 4b) of the resampled time series (also see Fig. S10). Therefore, calibration is necessary for the IOD product for models to represent a realistic IOD strength.

## Standard operating procedure (SOP) for the IOD warning system

In this section, we consolidate the choices made for the IOD Warning System based on the analysis above in Table 4. The warning system will use DMI to monitor and predict the IOD. We have also considered the application of the eastern pole of the IOD (referred to as SETIO) for rainfall prediction in Southeast Asia due to its proximity to the region. However, we found that SETIO shows a similar magnitude of correlation with rainfall over Southeast Asia and, therefore, does not offer any substantial advantage over DMI. Nevertheless, the DMI forecasts will be complemented with SETIO and WTIO forecasts to monitor the SST gradients and identify the pseudo-IOD events (see below in this section).

It is important to note here that simply using an index like DMI for the monitoring of the IOD, and associated impacts on hydrometeorological conditions, can lead to false alarms or missed events due to errors in observations, errors in the model predictions as well as due to the fact that some regions in Southeast Asia can experience the impact of the IOD before meeting the operational criterion laid out in Table 3. Therefore, for a robust warning system, we also consider continually monitoring the real-time physical conditions, e.g., outgoing longwave radiation (Fig. S11) to issue the IOD alerts, alongside monitoring other drivers that affect the rainfall in the region.

There are also impacts of climate change on the Indian Ocean basin that would need to be considered. The DMI is an index based on the SST-anomaly gradient between the western and the eastern Indian Ocean, which partially cancels the impact of climate change on the Indian Ocean basin. However, if the impact is uneven across the basin, for example, both the western and eastern Indian oceans are warmer than normal but the magnitude of warming differs, it can potentially lead to pseudo-IODs (e.g., paper^12^. It is not well understood if the impacts of the pseudo-IOD on rainfall and other climate variables are similar to those of the typical IOD events, therefore, for the IOD Warning System, we consider monitoring these events but flagging them separately.

**Fig. 5:**
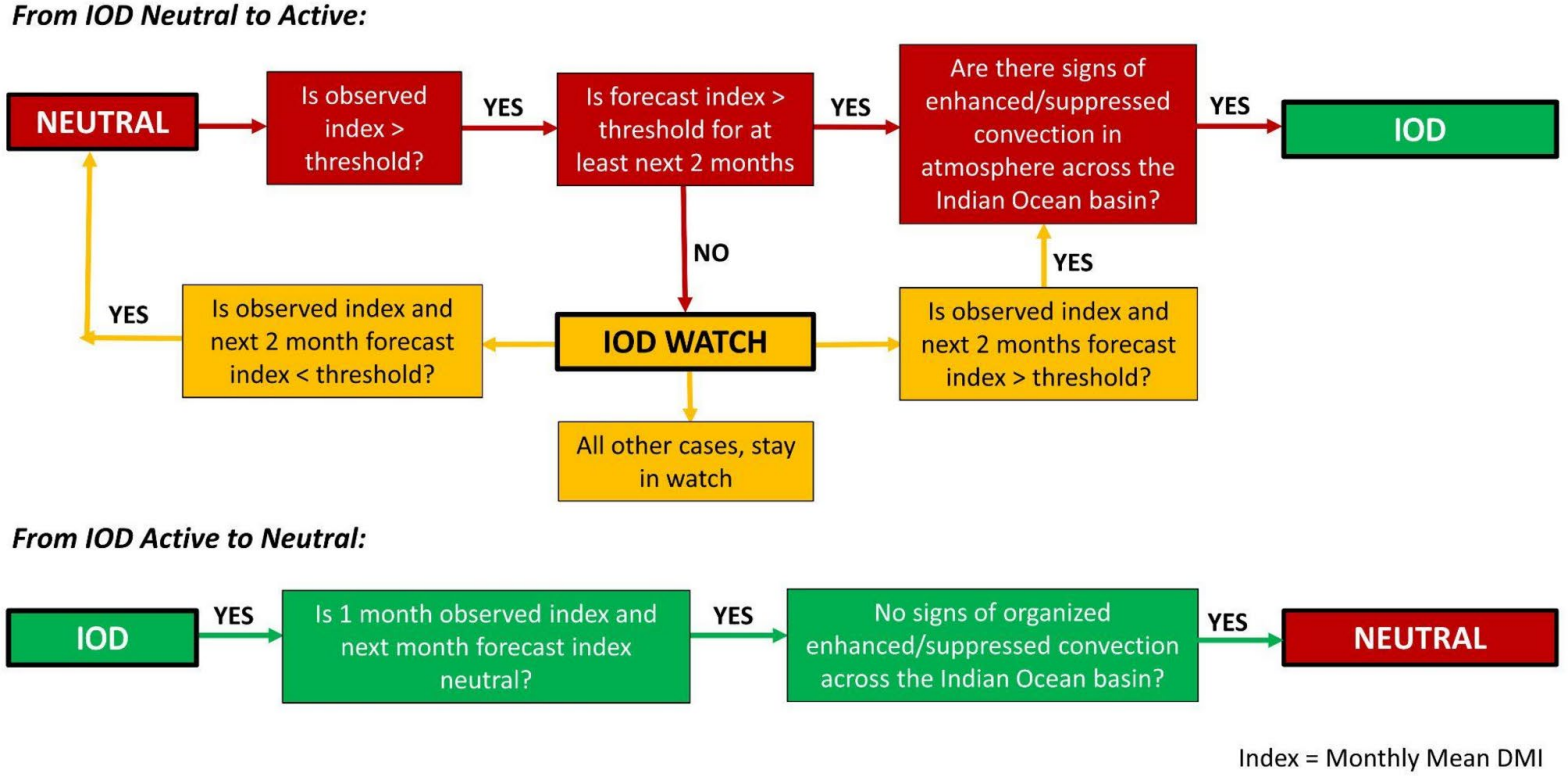
Standard Operating Procedure (SOP) to assist climate forecastors in issuing the IOD warnings and deciding the state of the IOD.

We also provide the SOP for the IOD Warning System to assist climate forecasters in making an objective assessment of the state of the IOD and issuing warnings of IOD events (Fig. 5). The start, duration, and end of the pIOD and nIOD events in the historical record based on the above criterion are shown in Fig. S4 (Supplementary material).

We also highlight here not only the phase and magnitude of the IOD event is important but the timing of the IOD event is also equally important for hydrometeorological hazards in Southeast Asia. For example, if there is a pIOD during JJAS, similar to the 2019 and 2023 pIOD events, it can lead to exceptionally dry conditions and exacerbate the risk of drought over the Maritime Continent. Whereas a pIOD in October–November would counter the wet conditions over the same region. We provide a summary of the impact of the pIOD and nIOD phases for different seasons and regions in Table 4.

**Table 3:** Summary of regions that experience significant IOD impacts. Only those regions are shown for which the correlations are significant (Fig. 2).

S.No.	Region	Period	Rainfall characteristic of the selected region for the given period	Phase of IOD	Impact on the region
1.	Maritime continent	JJAS	Relatively dry	pIOD	Dry getting drier
				nIOD	Dry getting wetter
2.	Maritime continent	ON	Relatively wet	pIOD	Wet getting drier
				nIOD	Wet getting wetter
3.	Eastern Mainland Southeast Asia	ON	Relatively wet	pIOD	Wet getting drier
				nIOD	Wet getting wetter
4.	Philippines	ON	Relatively dry	pIOD	Dry getting drier
				nIOD	Dry getting wetter

**Table 4:** Details of the proposed operational IOD warning system for Southeast Asia.

Variable	Real-time	Historical record	Reasoning
SST dataset	Mean of COBE, HadISST, ERSST (or whichever is available)	Mean of COBE, HadISST, ERSST	Using multi-observational mean DMI reduces the statistical noise in the DMI time series and shows either comparable or better skill scores than individual SST observational datasets. The multi-observational mean DMI does not have any significant trend whereas individual SST datasets, e.g. HadISST, show a monotonically increasing trend in the DMI time series
Baseline climatology	1991–2020	1991–2020	In line with the WMO guidelines for climate normals (https://community.wmo.int/en/wmo-climatological-normals), and other operational products being produced by Southeast Asia Regional Climate Centre Network (demonstration phase)
Threshold	0.4	0.4	This value is close to the 1 SD in multi-observational mean DMI from June to December and in line with thresholds used by other operational meteorological centers Note that some regions in Southeast Asia, e.g., the Southern Maritime Continent, may experience the impacts of the IOD on rainfall for DMI less than the threshold value of ± 0.4, i.e. when the IOD has not met the operational criterion. Therefore, caution must be employed when issuing rainfall outlooks for these regions
Diagnostic	Monthly-mean DMI	3-month running mean DMI	As IOD events typically last for 3–6 months, using a 3-month running mean DMI may not immediately reflect the IOD event in real-time. For the historical events, using a 3-month running mean reduces the monthly variability due to other rainfall drivers (Fig. S2) Note that this can sometimes lead to disagreement in the real-time monitoring and retrospective evaluation of the IOD event, particularly for weak IOD events
Models	Multimodel ensemble	NA	Multimodel ensemble produces better skill scores (Fig S6)
IOD event criterion	Monthly mean DMI exceeding the threshold for at least 3-consecutive months	3-months running mean DMI exceeding the threshold for at least 3-consecutive months	As IOD events typically last for 3–6 months, we use a minimum of 3 consecutive months, for an event to start, peak and decline
Event period	The period between the earliest and latest month when the DMI meets the criterion	Same as real-time	Note that a year will be identified as an IOD year if an IOD event is observed in that year, e.g., for an IOD extending from September–January 2023, 2023 is mentioned as IOD year
Outlook calibration	Necessary for standard deviation	NA	Most models analyzed here have an overactive IOD strength and therefore calibration is necessary for more reliable predictions
Pseudo IOD	Same sign for SETIO and WTIO, but fulfill all other criteria of the IOD event	Same as real-time	The DMI is defined as the difference in the SST anomalies across the South Eastern Tropical Indian Ocean (SETIO) and Western Tropical Indian Ocean (WTIO). If both SETIO and WTIO have the same sign for the SST anomalies (e.g. both are warmer than average), but different magnitudes, DMI can still show substantial values
Atmospheric/oceanic Indicators	Supporting conditions in either OLR, upper ocean heat content, or 850 hPa or 200 hpa wind speed anomalies	Same as real-time	IOD is a coupled atmospheric-oceanic phenomenon and the impact of IOD on rainfall is manifested through the atmosphere. Therefore, the response of IOD in atmospheric and oceanic indicators is expected (e.g., Fig. S10)

## Conclusions and future outlook

In this paper, we did a stock-take of the operational products and criteria used for monitoring and prediction of IOD events by multiple modeling centers. While many operational centers use a DMI threshold of 0.4 to identify an IOD event, they use different SST observational datasets, baseline climatology, or choice of time-averaging (monthly vs. 3-monthly mean DMI) for the DMI. We find that the DMI is particularly sensitive to the SST observations and time-averaging, which can lead to marked differences between the conclusions of different operational centers on the present state as well as the outlook of the IOD. We also note that the DMI threshold is mainly chosen for its statistical relevance—a DMI of 0.4 is approximately equal to one standard deviation in the monthly DMI time series—rather than the impact on regional climate. However, some regions in Southeast Asia (e.g. Southern Maritime Continent) could experience the impacts of IOD on rainfall for a weak IOD event or even when IOD has not met the operational criterion, which could be due to the proximity to the eastern Indian Ocean basin and strong ocean-atmosphere coupling over this region. Multimodel forecast products providing a chance of exceeding different DMI thresholds (e.g. chance of DMI exceeding a threshold of 0.3, 0.4, and so on) could therefore be useful to take into account the diversity in the impact of IOD on different regions and support impact-based forecasting of the IOD.

We examined the performance of the latest seasonal prediction systems in capturing the phase and strength of the IOD. While most models are skillful in capturing the phase of the IOD for June-December (the period when IOD is usually observed), all models analyzed here have over-active IOD strength. Therefore, the calibration of the DMI-based monitoring products can yield more reliable predictions. All models have lower skill for the hindcasts initialized during January–May, likely due to inactive IOD between January–June. Therefore, caution must be exercised when issuing IOD forecasts initialized during this period. We also discussed the role of observational errors which could lead to apparently low skill scores. Using a multi-observational mean dataset for verification can yield better skill scores.

Finally, we outline the criterion and SOP for the IOD Warning System for early prediction of hydro-meteorological hazards in Southeast Asia, in line with WMO practice on an objective approach toward seasonal outlooks. It is worth noting here that the IOD Warning System is one of the tools that is useful in managing hydro-meteorological hazards over Southeast Asia, however, dynamical predictions of rainfall and temperatures, which may include the influence of many climate drivers in addition to the IOD, should also be considered, particularly for local outlooks. In this work, we have only explored the application of SST-based indices for the IOD warning system. However, it might be worth exploring the application of multivariate indices for this purpose. This work also highlights that subjective choices and decisions have a critical role to play in the development of any warning system. In this case, the development of the IOD warning system, including investigating the subjective decisions required, has allowed us to better understand the limitations of the system and the impacts of IOD events.

## Electronic supplementary material

Below is the link to the electronic supplementary material.

Supplementary Material 1

## Data Availability

All data used in this work is publicly available. The seasonal hindcast data was obtained through Copernicus Climate Change Services (C3S) Climate Datastore (https://climate.copernicus.eu/seasonal-forecasts), rainfall observations from FROGS (http://climate-cms.wikis.unsw.edu.au/FROGs), SSTs from COBE (https://psl.noaa.gov/data/gridded/data.cobe.html), ERSST (https://psl.noaa.gov/data/gridded/data.noaa.ersst.v5.html) and HadISST (https://www.metoffice.gov.uk/hadobs/hadisst/index.html).
