# Supplementary material for: Towards developing an operational Indian ocean dipole warning system for Southeast Asia
Source: Sci Rep. 2025 Apr 27;15:14728. doi: 10.1038/s41598-025-99261-9 (PMC12034776; doi:10.1038/s41598-025-99261-9)
Supplement: Supplementary file 1 — Supplementary Material 1 [file 41598_2025_99261_MOESM1_ESM.docx]

**Supplementary Material for**

**“Towards Developing an Operational Indian Ocean Dipole Warning System for Southeast Asia”**

Shipra Jain^1,2*^, Thea Turkington^2^, Wee Leng Tan^2^, Chen Schwartz^2^, Adam A Scaife^3,4^, and Theodore G Shepherd^5,6^

^1^Department of Risk and Disaster Reduction, University College London, United Kingdom

^2^Centre for Climate Research Singapore, Meteorological Service Singapore, Singapore

^3^Met Office Hadley Centre, Exeter, United Kingdom

^4^Faculty of Environment, Science and Economy, University of Exeter, Exeter, United Kingdom

^5^Department of Meteorology, University of Reading, Reading, United Kingdom

^6^Jülich Supercomputing Centre, Forschungszentrum Jülich, Jülich, Germany

*Corresponding Author, Email: shipra.jain@ucl.ac.uk, Tel: +44-7587437690This document contains supplementary material for the manuscript entitled “Towards Developing an Operational Indian Ocean Dipole Warning System for Southeast Asia”. This material contains Tables S1-S2 and Figures S1-S11 cited in the main text.

**Table S1.** Set of 16 different definitions used for identifying IOD years. All definitions below use a threshold of DMI = 0.4 for three consecutive months to identify the year as a pIOD or nIOD year.

| **S.No** | **IOD Definition** | **SST Dataset** | **Baseline Period** | **Time Averaging used for the Index** |
| --- | --- | --- | --- | --- |
| 1 | COBE1 | COBE-2 | 1981-2010 | Monthly Mean |
| 2 | COBE2 |  | 1991-2020 | Monthly Mean |
| 3 | COBE3 |  | 1981-2010 | 3-Month Running Mean |
| 4 | COBE4 |  | 1991-2020 | 3-Month Running Mean |
| 5 | ERSST1 | ERSST | 1981-2010 | Monthly Mean |
| 6 | ERSST2 |  | 1991-2020 | Monthly Mean |
| 7 | ERSST3 |  | 1981-2010 | 3-Month Running Mean |
| 8 | ERSST4 |  | 1991-2020 | 3-Month Running Mean |
| 9 | HadISST1 | HadISST | 1981-2010 | Monthly Mean |
| 10 | HadISST2 |  | 1991-2020 | Monthly Mean |
| 11 | HadISST3 |  | 1981-2010 | 3-Month Running Mean |
| 12 | HadISST4 |  | 1991-2020 | 3-Month Running Mean |
| 13 | ObsMean1 | Mean of COBE, HadISST, and ERSST | 1981-2010 | Monthly Mean |
| 14 | ObsMean2 |  | 1991-2020 | Monthly Mean |
| 15 | ObsMean3 |  | 1981-2010 | 3-Month Running Mean |
| 16 | ObsMean4 |  | 1991-2020 | 3-Month Running Mean |

**Table S2**: Operational center websites from where the information on IOD product is taken

| **Centre** | **Website** | **Last Accessed** |
| --- | --- | --- |
| APEC Climate Centre (APCC) | https://apcc21.org/ser/enso.do?lang=en  https://apcc21.org/ser/indic.do?lang=en | 24/12/2023 |
| Bureau of Meteorology (BoM) | <http://www.bom.gov.au/climate/enso/#tabs=Indian-Ocean>  and Personal communication | 24/12/2023 |
| Copernicus Climate Change Service (C3S) | https://climate.copernicus.eu/charts/packages/c3s_seasonal/products/c3s_seasonal_plume_mm?area=iod&base_time=202304010000&type=plume | 24/10/2024 |
| Indian National Centre for Ocean Information Services (INCOIS) | https://incois.gov.in/portal/IOD | 24/10/2024 |
| Japan Meteorological Administration (JMA) | https://ds.data.jma.go.jp/tcc/tcc/products/elnino/iodevents.html  <https://ds.data.jma.go.jp/tcc/tcc/products/elnino/index/iod_index.html> | 24/12/2023 |
| National Oceanic and Aeronautics Administration (NOAA) | https://psl.noaa.gov/gcos_wgsp/Timeseries/DMI/ | 24/12/2023 |
| UKMO | https://www.metoffice.gov.uk/research/climate/seasonal-to-decadal/gpc-outlooks/atlantic-indian-ocean  and personal communication | 24/12/2023 |
| World Meteorological Organization Lead Centre | https://www.wmolc.org/contents/index/Climate+Indices  and personal communication | 24/12/2023 |

**
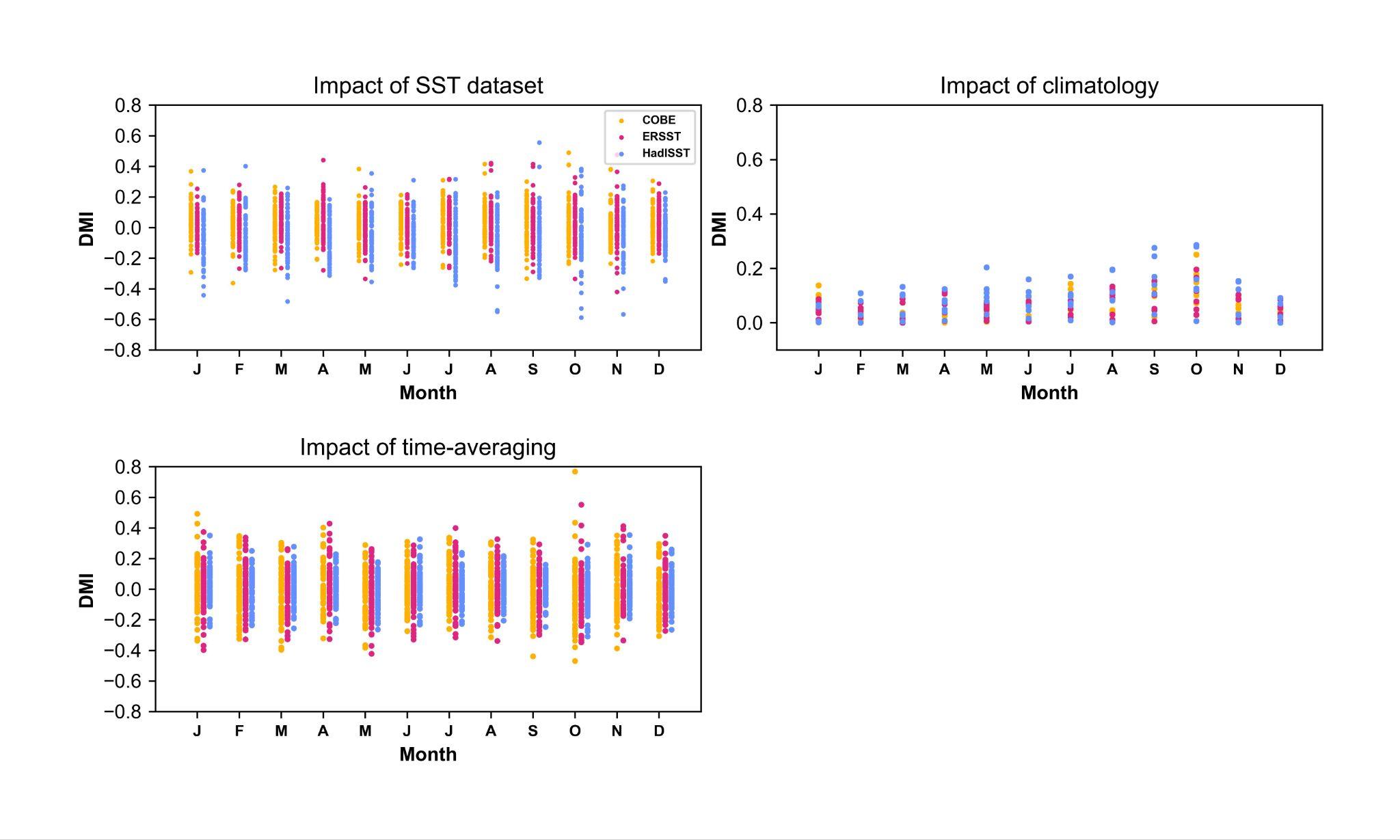
**

**Fig. S1 (a)** Difference in the monthly mean DMI between the individual SST dataset and the multi-observational mean DMI (1991-2020 climatology used for all SST datasets) **(b)** Absolute difference in the monthly DMI between 4 different climatological periods (1961-1990, 1971-2000, 1981-2010, 1991-2020) **(c)** Difference between monthly mean and 3-month running mean DMI (using 1991-2020 climatology). All panels are for the monthly DMI values for 1960-2021.


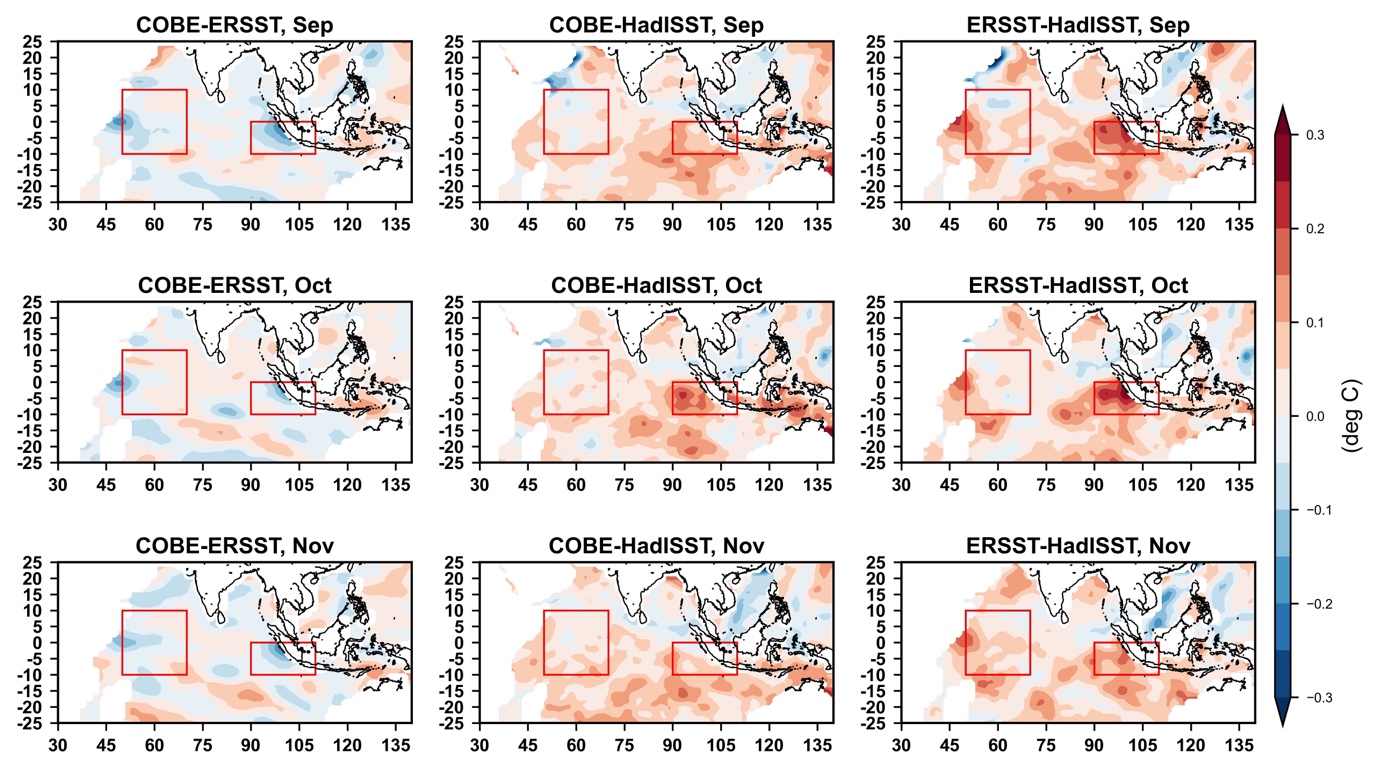


**Fig. S2** Difference in standard deviation between COBE, ERSST, and HadISST for Sep-Nov using SST data from 1960-2021.


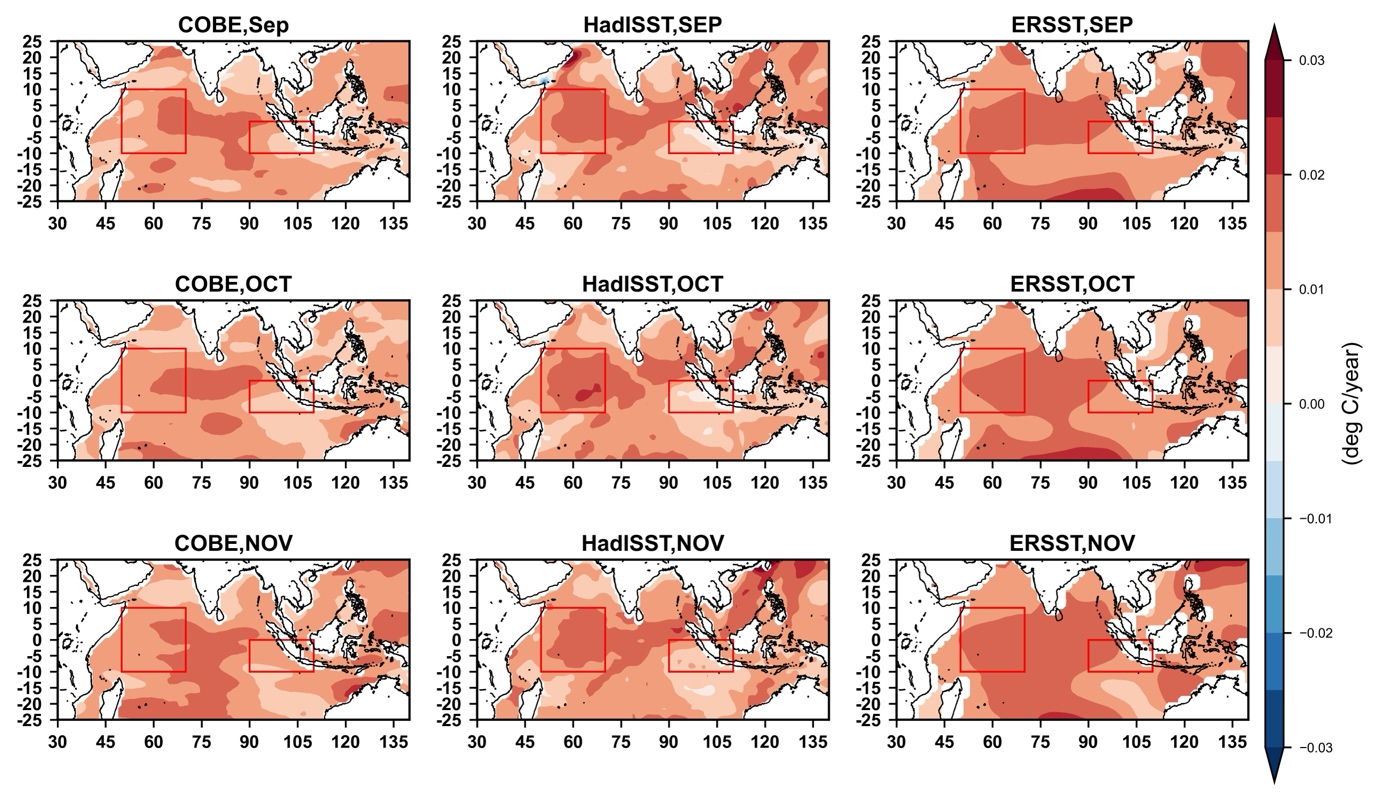


**Fig. S3** Trend in SST in COBE, HadISST, and ERSST for autumn months (September to November). The trends are calculated for 1960-2021.

**
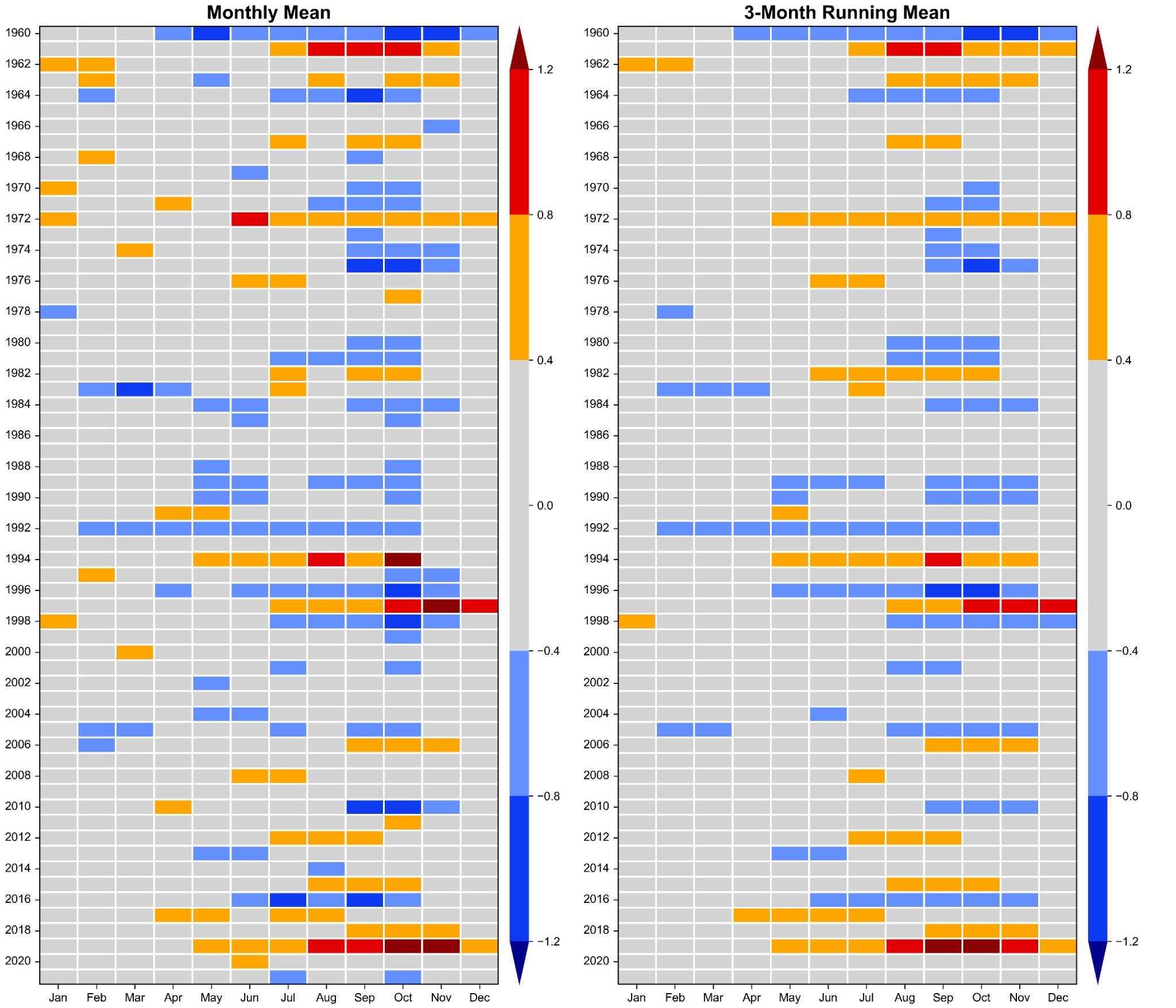
**

**Fig S4 (a)** Monthly mean DMI and **(b)** 3-month running mean DMI using multi-observational mean and baseline climatology of 1991-2020 for the historical record. The DMI threshold of 0.4 is used to identify an IOD event.
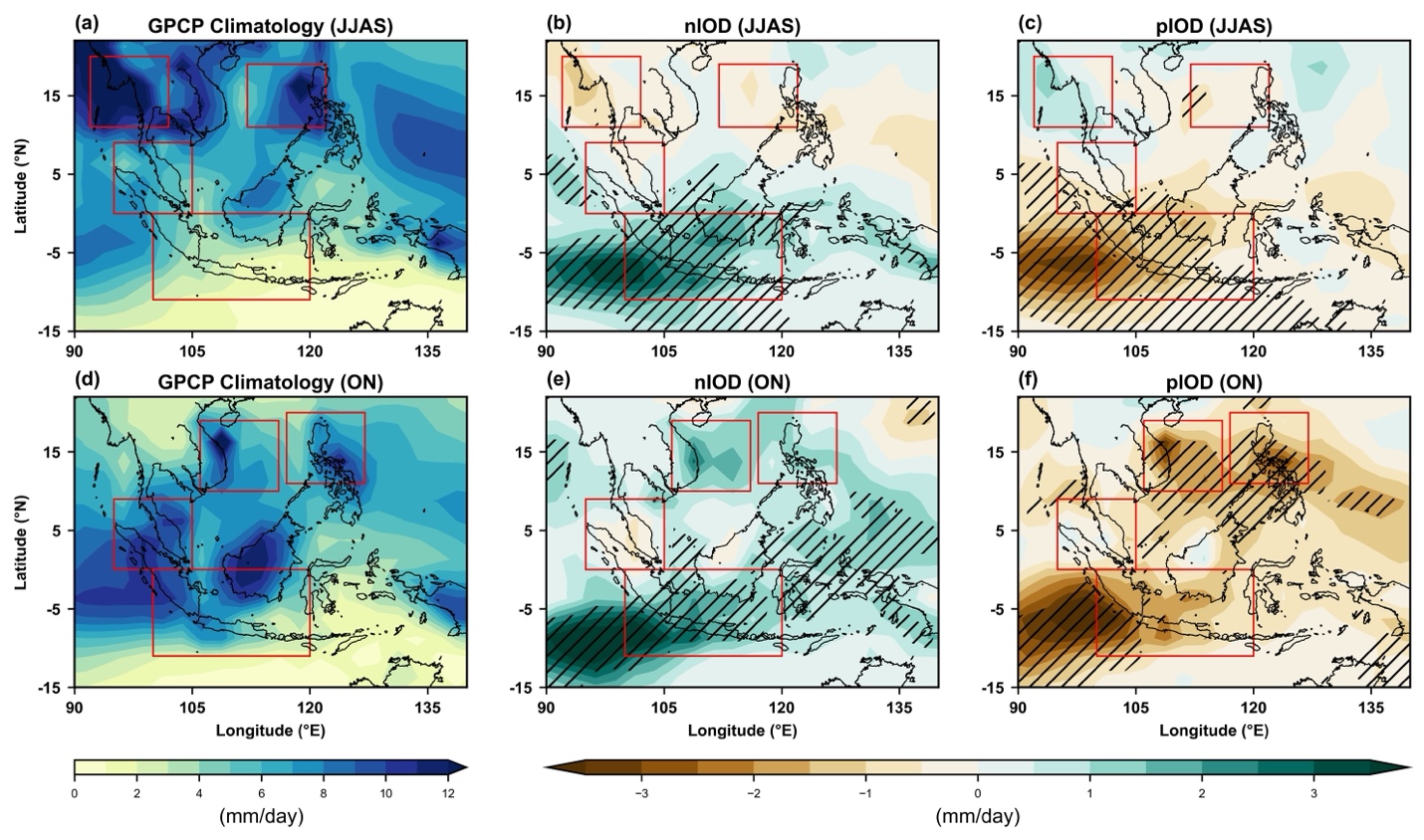
**Fig. S5 IOD and rainfall over Southeast Asia** Mean rainfall climatology for 1979-2021 from the GPCP for **(a)** June-September (JJAS) **(d)** and October-November (ON). The composite rainfall anomalies (mm/day) for the nIOD and pIOD are shown for **(b-c)** JJAS and **(e-f)** ON. The rainfall anomalies are calculated with respect to the corresponding 1979-2021 mean climatology. Hatched regions show areas that show statistically significant rainfall differences with respect to climatology. The pIOD and nIOD years are identified using multi-observational mean DMI with respect to the 1979-2021 climatology (same as rainfall climatology) using the DMI threshold of 0.4. For nIOD, 9 years were used (1981, 1984, 1989, 1992, 1996, 1998, 2005, 2010, 2016) and for pIOD, 11 years were used (1982, 1983, 1994, 1997, 2006, 2008, 2012, 2015, 2017, 2018, 2019) for composites. The latitude, longitude and naming convention of the spatially aggregated regions are as follows: Southern Maritime Continent (100-120 °E, -11-0 °N), Western Maritime Continent (95-105 °E, 0-8 °N), Western Mainland Southeast Asia (92-102 °E, 11-20 °N), Eastern Mainland Southeast Asia (106-116 °E, 10-19 °N), South China Sea (112-122 °E, 11-19 °N) and Philippines (117-127 °E, 11-20 °N). The regions selected for spatial aggregation are based on the response of rainfall to the two IOD phases and also climatological mean rainfall for that season are shown by black boxes. We focus on land regions rather than the ocean and we chose June-November as most IOD events develop and peak during this time of the year. We use the same base climatology for DMI as rainfall (1979-2021) for a one-to-one comparison.


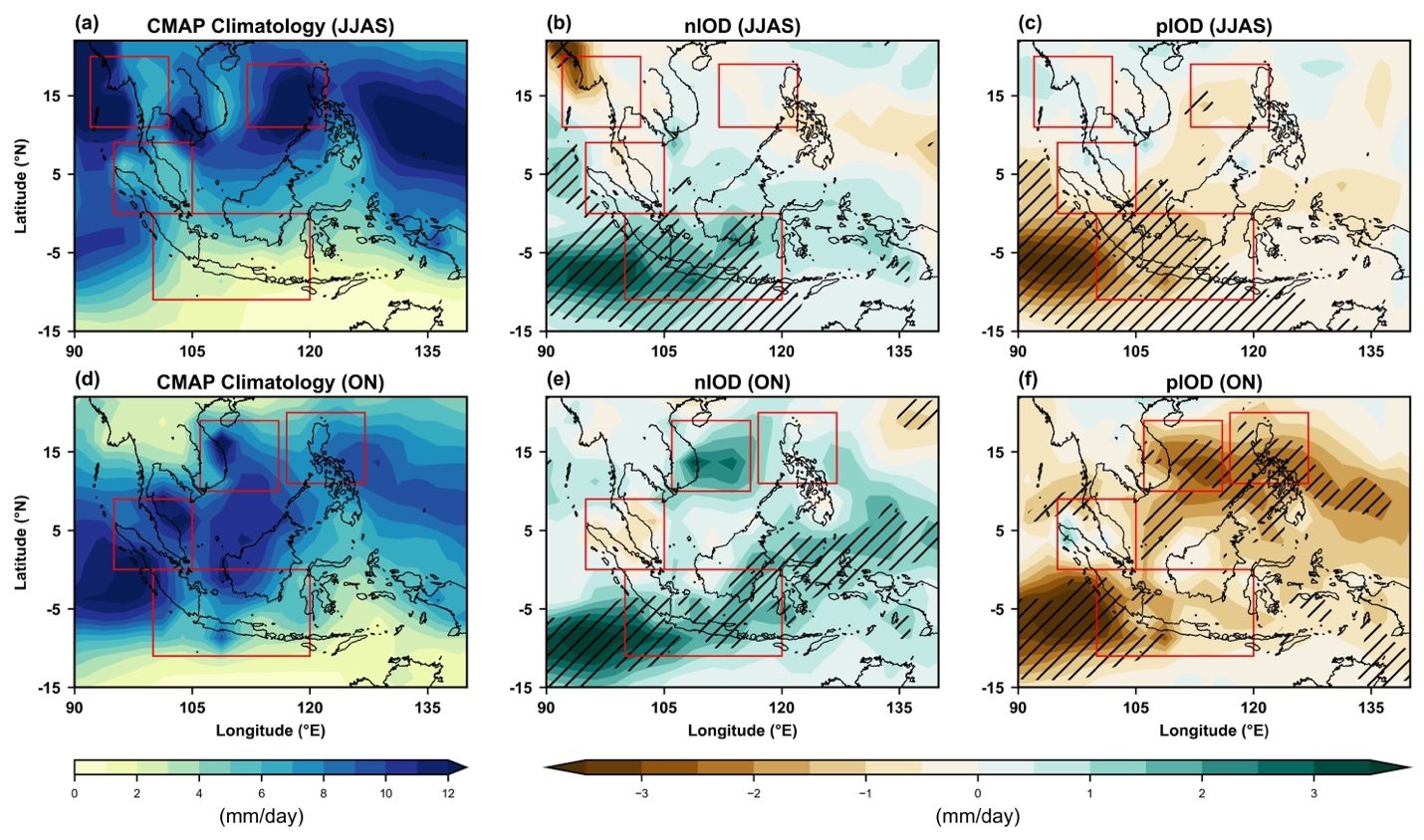


**Fig. S6 IOD and rainfall over Southeast Asia** Same as Fig. S5 but for CMAP rainfall.


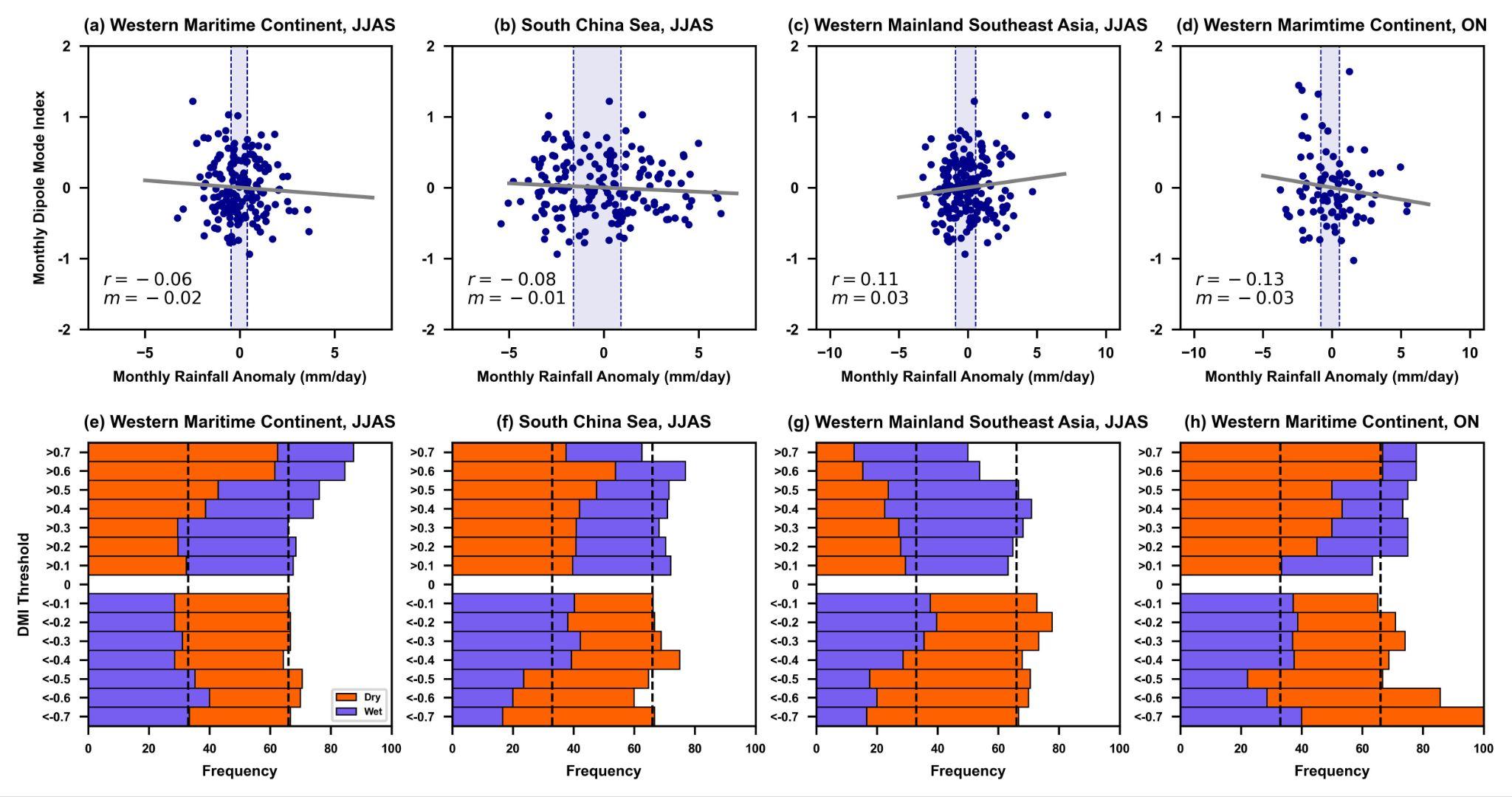


**Fig. S7 Observed relationship between regional rainfall and IOD phases.** Same as Fig. 3 but for combinations of regions and seasons for which the correlations were insignificant.


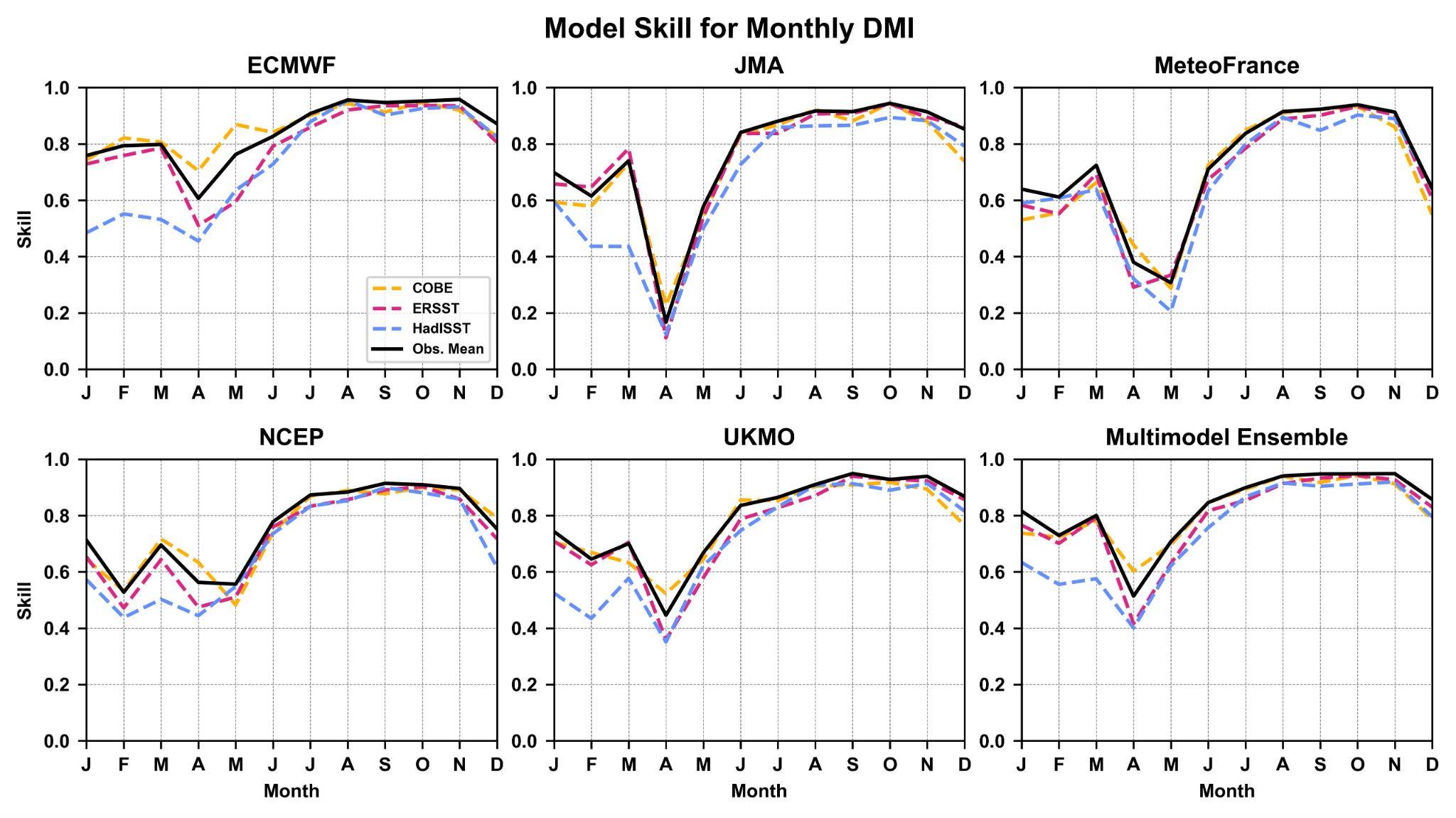


**Fig. S8 Model skill in capturing the phase of the IOD.** The skill scores for DMI time series using model hindcasts for one month lead time and for the period, i.e. 1993-2016, using three different observational datasets (colored dashed lines) and multi-observational mean (solid black line). For UKMO, the hindcast data is not available for January 1993 and therefore the skill is calculated for 1994-2016 for January. Multimodel ensemble skill is calculated by averaging the ensemble mean DMI time series of all models, and then correlating it with the observational time series.


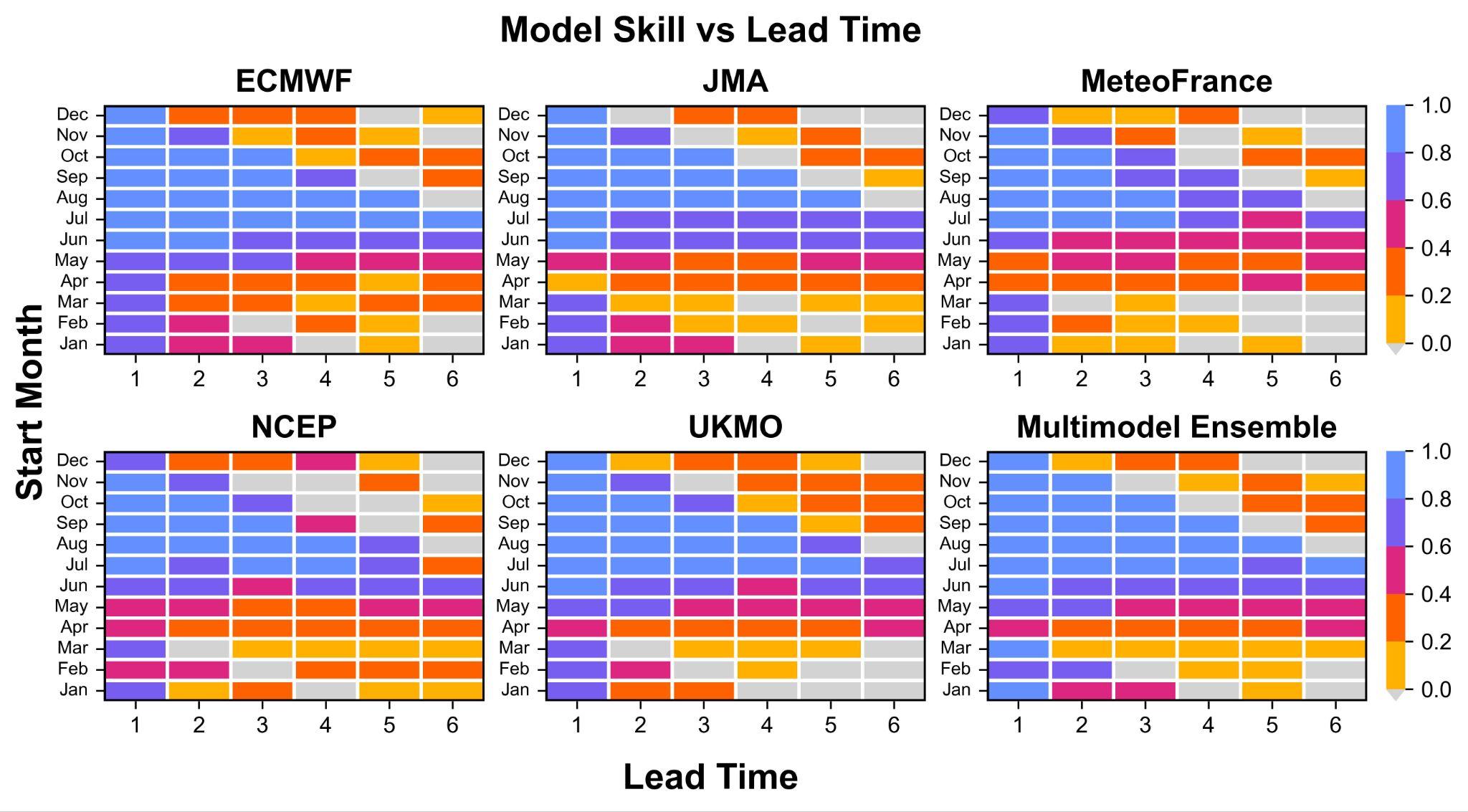


**Fig. S9 Model skill and lead times** Model skill scores for common hindcast period, 1993-2016, and different lead times. The skill scores are calculated against the multi-observational mean. Multimodel ensemble skill is calculated by averaging the ensemble mean DMI time series of all models, and then correlating it with the observational time series.


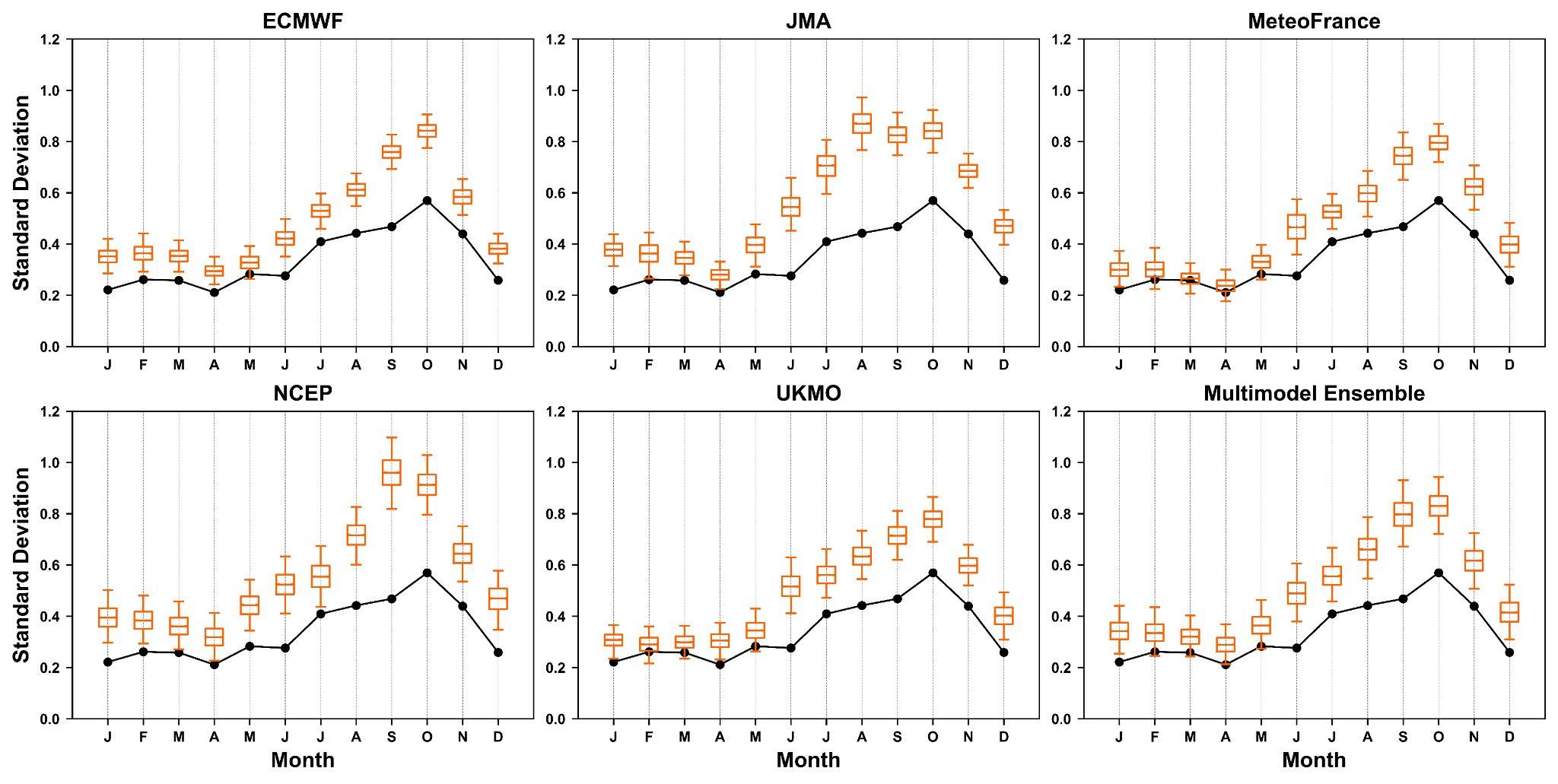


**Fig. S10 Unprecedented Simulated Extremes using Ensembles (UNSEEN) test**^27^ **for individual models.** Same as 5b but for individual models as well as multimodel ensembles.


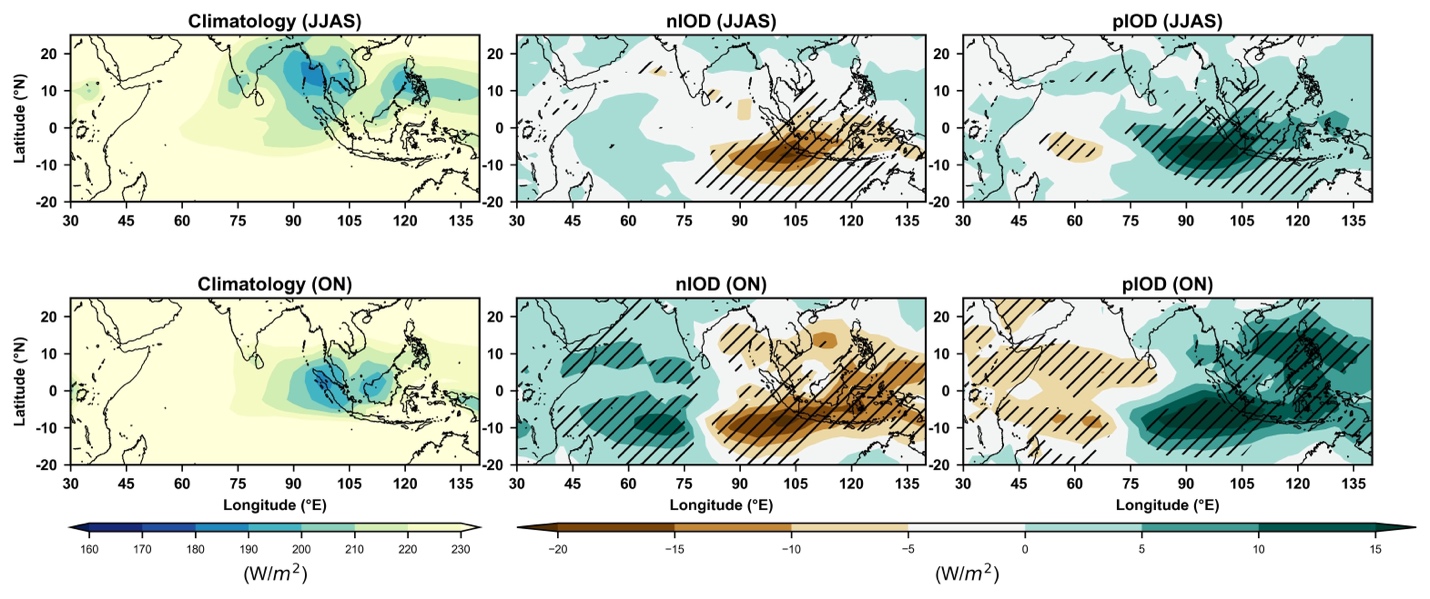


**Fig. S11 Atmospheric indicator of the IOD** Mean Outgoing Longwave Radiation (OLR, Wm^-2^) for 1979-2021 for JJAS and ON. OLR anomalies for nIOD years and pIOD years with respect to 1979-2021 climatology. The years used for nIOD and pIOD are the same as for Fig. 2. Hatched regions show areas that show statistically significant OLR differences with respect to climatology.
